# Family participation to enhance care and tackle health worker shortages in resource-limited hospitals: A systematic review

**DOI:** 10.7189/13.04005

**Published:** 2023-01-20

**Authors:** Jim J Determeijer, Stije J Leopold, René Spijker, Charles Agyemang, Michèle van Vugt

**Affiliations:** 1 Department of Infectious disease, Amsterdam University Medical Centers, Amsterdam, the Netherlands; 2 Department of Public Health, Amsterdam University Medical Centers, Amsterdam, the Netherlands; 3 Medical Library, Amsterdam University Medical Centers, Amsterdam, the Netherlands

## Abstract

**Background:**

A growing global shortage of health workers is limiting access to health care, especially in resource-limited countries. Family participation in hospital care could enhance care while tackling health worker shortages. With the same resources, it might deliver additional and more personalised care. This review assessed the effect and safety of family participation interventions in the care of hospitalised adults in resource-limited settings and, ultimately, if it is a viable strategy to tackle health worker shortages.

**Methods:**

For this systematic review, Medline, Embase, CINAHL and the Global Health Library were searched from inception till April 7, 2022. Clinical studies were included if they described a family participation intervention for hospitalised adults, were performed in a low- or middle-income country and reported on a patient-related outcome. Data were collected on patient, family, staff and health service-related outcomes. Risk of bias was assessed with the ROB2 and ROBINS-I tool.

**Results:**

From 4444 studies, six were included for narrative synthesis, with a total of 1794 participants. Four studies were performed in Asia and two in Africa; all were published between 2017 and 2022. In-hospital family participation interventions aimed at medication administration and adherence, delirium prevention, and palliative cancer care were successful in significantly improving patient outcomes. Involving family in post-stroke rehabilitation interventions showed no significant effect on mortality and long-term disability. Few data were reported on participating family members’ outcomes or hospital staffing issues. None of the included studies showed harm from family participation.

**Conclusions:**

The limited data suggest that family participation can be effective and safe in specific contexts. However, more research is needed to determine the effect of family participation and justify further implementation. Family participation research for enhancing care while tackling health worker shortages should be a collaborative priority of researchers, health care professionals, funding agencies and policymakers.

**Registration:**

PROSPERO registration No. CRD42020205878.

A shortage of health workers remains a major obstacle to global universal health care coverage. The World Health Organization (WHO) reported a global shortage of 7.2 million health workers in 2013, with 83 countries facing a health worker crisis [1]. The WHO projected an additional 18 million health workers to be needed by 2030 [2], requiring at least six million nurses [3]. Healthcare systems most affected by this crisis are in resource-limited countries [1]. Therefore, in addition to existing strategies, innovative approaches are urgently needed to cope with shortages in health care personnel.

In understaffed hospitals in resource-limited countries, family members often fill the gap by ensuring basic care for their admitted relatives. For example, family members may feed, wash, administer drugs, treat wounds or alert doctors and nurses to clinical deterioration and medical emergencies. However, in most circumstances, the family caregiver role is informal, unsupported and untrained, while the burden of care is high and the knowledge of hospital hazards is poor [4-16]. Family participation in these environments is necessity driven and informally shifts tasks from (para)medical staff to family members. In comparison, family participation in resource-rich countries’ hospitals has successfully enhanced staff-delivered care with an emphasis on emotional support, accelerated recovery or improved discharge preparation instead of tackling health worker shortages [17-21].

Supporting and training family members to participate in basic care in resource-limited hospitals could provide a safe and effective opportunity to enhance care while tackling health worker shortages. By selectively shifting tasks from health care staff, for example nurses, to family, with staff in a coordinating and educating role, more care could be delivered with the same resources. Additionally, family-delivered care could stimulate personalised care with, for example, quicker recovery, fewer adverse events and higher quality of life. From the family’s perspective, support and training for participation could lower their current burden of informal hospital care and prepare them for the care at home when the patient is discharged. Finally, for the staff and health service, formalised family participation could free up time and lower stress on understaffed care facilities.

However, whether family participation in resource-limited care is safe and effective remains uncertain. Previous research has been performed in high-resource countries, home-based care and paediatrics, yet no systematic reviews have been performed on family participation in resource-limited hospital care for adults. Therefore, this systematic review aims to assess the effect and safety of family participation on patient, family, staff and health service outcomes in the care of hospitalised adults in resource-limited countries and, ultimately, if it is a viable strategy to tackle health worker shortages.

## METHODS

### Protocol and registration

Before starting this systematic review, a study protocol was established following the PRISMA guidelines [22]. Subsequently, the review was registered on October 15, 2020 in the PROSPERO database under CRD42020205878 [23]. There are no differences between the protocol and the review.

### Family participation definition

The field of research this systematic review aims to cover is largely undefined. Many seemingly synonymous terms have been used in publications to refer to overlapping and distinctively different concepts and definitions. No consensus exists on the correct use of these terms. The term “family participation” was chosen as it is the most literal description of the intervention type intended to be included in this review and closest to the definition used by the Institute for Patient- and Family-Centered Care [24]. In this review, “family participation” means the active participation, instruction-guided, of a family member or close relative in the delivery of care by executing care tasks for their relative.

### Eligibility criteria

#### Types of studies

All interventional studies, randomised and non-randomised, assessing the effects of a family participation intervention aimed at improving patient, family, staff or health service outcomes among hospitalised adults were included. Studies had to report on at least one patient outcome to be included. Observational studies describing a status quo or interventions without reporting any patient outcome measure were excluded. Publications in English, Dutch or French were included. Other languages were excluded after assessing relevance in title and abstract using web-based translation. Case reports, correspondence, reviews, conference abstracts and unpublished data were excluded. A study was also excluded if the abstract or full text was unavailable from online databases.

#### Types of participants

The patient participants had to be adults and admitted to an adult ward. The definition of “adult” may vary by region, but we considered patients above 16 years as adults in this review. The caregiver participating in the intervention had to be a family caregiver or have a personal connection to the patient. In some societies, the extended family does not involve direct family ties but does contribute strongly to the “family” structure in that culture. Therefore, hired or volunteering attendants without a personal connection to the patient were excluded for this systematic review.

#### Types of environments

Included were interventions conducted in a hospital-like environment, such as hospitals, hospices and nursing homes. In resource-limited countries, the differences between these environments can be small, and a similar family participation dynamic exists where care is delivered in collaboration by staff and the participating family.

The intention was to include studies performed in resource-limited settings. In this review, resource-limited settings were defined as low-, lower-middle- and upper-middle-income economies according to the World Bank criteria [25]. Upper-middle-income economies were also included as the WHO states that only 52% of these economies are above a threshold of 22.80 health care workers per 10 000 population. This threshold is considered the minimum to run essential health care services [1]. Resource inequalities between hospitals exist within a country, but it is impossible to judge each health service’s individual resource limitations within a study. Therefore, the World Bank criteria were used as an approximation with clear cut-off points.

### Outcome measures

The primary outcome of this review was the effect of family participation interventions on patient outcomes: mortality, adverse events, length of stay, readmission rate, disability and dependence, symptom severity and quality of life.

Secondary outcomes included family-related outcomes: incentives, facilitators, burdens and barriers to participating in care; staff-related outcomes: perceptions and burdens of family participation; and health service-related outcomes: cost of intervention and needed hospital resources.

### Information sources and search

A systematic literature search was compiled for the online databases Medline, Embase, CINAHL and the Global Health Library from inception till April 7, 2022. The search was built by an experienced clinical librarian (RS) using the keywords “family OR lay”, “caregiver”, “hospital” and “resource-limited setting” and expanded using synonyms or database tools such as MESH terms. No language restrictions were imposed in the search. In addition, the searches were supplemented by manually checking reference lists of relevant publications. The full search queries can be found in Supplement 1 in the **Online Supplementary Document**.

### Study selection

After duplicates were removed, all articles found were screened on the title, abstract and full-text, respectively, by two independent authors (JD, SL) using the eligibility criteria. Any conflicts in screening outcomes were discussed and agreed upon by the two initial reviewers and a third reviewer (MvV). The web application Rayyan was used for blind and independent screening [26].

### Data collection

After screening, data were extracted from the included articles into an excel sheet with predefined data items. Due to the explorative nature of this review, additional descriptive data for patient, family, staff and health service outcomes were gathered. One individual (JD) performed data extraction, and any uncertainties during data collection were discussed with the second and third reviewers (SL, MvV).

### Risk of bias in individual studies

All randomised controlled trials were assessed on their risk of bias using the Revised Cochrane Risk-Of-Bias tool for randomised trials (ROB2) [27]. Non-randomised studies were assessed using the Risk Of Bias In Non-randomised Studies of Interventions (ROBINS-I) tool [28]. The results of the critical appraisal were visualised with the ROBVIS tool [29].

### Data synthesis

A narrative synthesis was performed of the included studies. The interpretations were supported by quantitative and descriptive data from individual studies. However, the data was unsuitable for statistical pooling or meta-analysis due to the heterogeneous nature of the included studies.

## RESULTS

**Figure 1:**
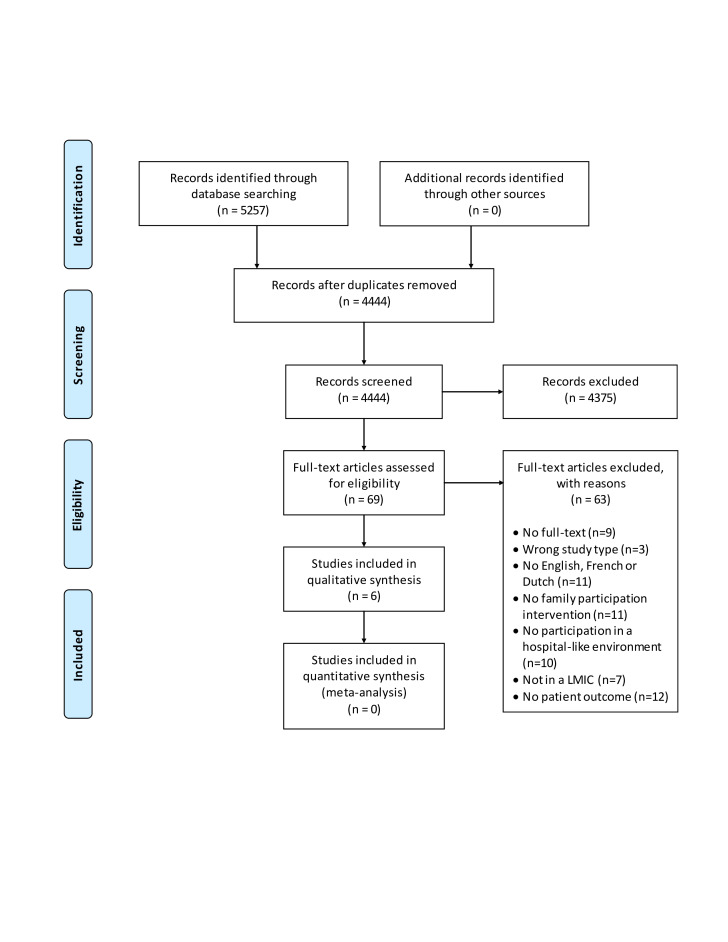
PRISMA screening flow-chart.

The literature searches resulted in 5257 records; after duplicates were removed, 4444 unique articles remained. Following title and abstract screening, 69 articles were considered eligible for full-text screening. Of these, six articles met the inclusion criteria. Reference checks resulted in no additional eligible studies. Studies excluded on language showed no relevance in title and abstract when assessed using web-based translation. A total of six articles were eligible for narrative analysis [30-35] (**Figure 1**). For the detailed list of excluded studies during full-text screening including exclusion reasons see Supplement 2 in the **Online Supplementary Document**

Of the included articles, four studies were performed in Asia and two in Sub-Saharan Africa. All studies were published between 2017 and 2022. The articles were heterogeneous regarding design, participants, intervention and outcomes. An overview of study characteristics and outcomes can be found in **Table 1**.

**Table 1:** Study characteristics and outcomes

Authors	Country	Publication year	Design	n (1794)	Participants’ condition	Family intervention*	Control	Primary outcome	Description of main outcome†
**Lindley et al.** [30]	India	2017	RCT	1212	Stroke	Physiotherapy training	Hospital physiotherapy	Dead or dependent	No difference between intervention and control regarding mortality, dependency, adverse events, length of stay, readmission or caregiver strain.
**Zhou et al.** [31]	China	2019	RCT	246	Stroke	Physiotherapy training	Usual care without rehabilitation instructions	ADL physical function	No difference between intervention and control regarding Barthel Index scores, length of stay, quality of life, caregiver burden and expenses in hospital.
**Wang et al.** [32]	China	2020	Cluster-RCT	281	Post-surgery, elderly	Delirium prevention training	Standard care, basic delirium care	Delirium rates	The intervention group had lower delirium rates during hospitalisation (NNT 5.9), less ADL decline, better cognitive status at discharge, shorter length of stay and better recovery after discharge.
**Kristanti et al.** [33]	Indonesia	2017	Cohort quasi-experimental	30	Cancer	Palliative care basic skills training	Baseline data	Quality of life	Compared to baseline, the quality of life improved, emotional and social functioning improved, and most symptoms after discharge decreased.
**Alupo et al.** [34]	Uganda	2017	QI	Not stated	Infectious diseases	Family education on their role in medication administration	Baseline data	Medication adherence	Compared to baseline, initial medication adherence improved from 46.5% to 98.0%; the follow-up rates variated from 80% to 95%.
**Wong et al.** [35]	Uganda	2022	QI	25	Undergoing gynaecologic oncology surgery	Perioperative fundamental care training	Baseline data	Adherence to perioperative procedures	No postoperative infections at discharge in either intervention or baseline group. Increased education on wound care (0% to 80%) and postoperative expectations (0% to 60%). No increase in caregiver documentation.

RCT – randomised controlled trial, QI – quality improvement, NNT – number needed to treat, ADL – activities of daily living

*All interventions were aimed at the attending family member and requested their participation in providing hospital care.

†Any differences between intervention and control or follow-up and baseline reported in this table were classified as significant in the respective study.

All family members received some form of individual [30-33,35] or individual and group [34] training. All interventions started during the hospitalisation of the patients, with three studies continuing the intervention after discharge [30,31,33] and four reporting follow-up data on the patients after discharge [30-33]. The follow-up ranged from up to hospital discharge to 6 months total.

As the studied conditions varied, so did the primary outcomes to suit the respective condition. Following the inclusion criteria, all studies reported at least one patient outcome. None of the studies reported on all the predefined data items, and only a few of the data items could be found in the studies. For an overview of the reported data items per study see Supplement 3 in the **Online Supplementary Document**. Often other patient, family, staff or health-service-related outcomes were reported than the ones predefined for this review.

The risk of bias for the three randomised controlled studies was assessed using the ROB2 tool. All scored a low risk of bias. The three non-randomised studies were assessed using the ROBINS-I tool. All scored a critical risk of bias due to major baseline and time-varying confounder risks, lack of study design information, deviation from the initial study design and unblinded outcome assessment. The detailed outcomes of the critical appraisal, visualised with the ROBVIS tool, can be found in Supplement 4 in the **Online Supplementary Document**.

As highlighted in **Table 1** and Supplement 3 in the **Online Supplementary Document**, there is little overlap between the reported outcomes of the studies. Overall, in-hospital family participation programs involving medication administration and adherence, delirium prevention, and palliative cancer care were successful in significantly improving patient outcomes. Involving family members in post-stroke rehabilitation interventions showed no significant effect on mortality and long-term disability. Few data were reported on participating family members’ outcomes or hospital staffing issues. None of the included studies showed any harm from family participation.

Regarding the primary patient outcomes of the studies, the studies including patients with stroke reported no significant difference between family-delivered rehabilitation, delivered in hospital and at home, and usual hospital rehabilitation on any measured outcomes. Of these studies, Lindley et al. described the same death or dependency numbers at six months of follow-up, odds ratio (OR) 0.98 (95% confidence interval (CI) = 0.78 to 1.23), *P* = 0.87 [30]. The other stroke-related study by Zhou et al. reported similar Barthel Index scores in the intervention and control groups, 70.10 ± 25.50 and 74.10 ± 23.00, (95% CI = -10.00 to 2.90), adjusted *P* = 0.27 [31]. The study by Wang et al. reported delirium within seven days post-surgery in four of 152 cases in the family-delivered prevention group compared with 25 of 129 cases in the staff-delivered prevention group (NNT 5.9) [32]. Kristanti et al. compared pre-and post-training palliative care skills and reported an improvement in the patients’ global health status from 40.27 (±17.79) to 56.94 (±18.05) on the EORTC QLQ C30 questionnaire (*P* = 0.001) [33]. The study of Alupo et al. described increased medication adherence, measured by sampling random patient files, from 46.50% to 98%, with fluctuating follow-up results from 80 to 95% through iterative PDSA cycles. In these PDSA cycles, interventions were implemented in parallel aimed at both family, staff and pharmacy. No differentiation was made in the outcomes to address the effect size of each intervention [34]. Finally, Wong et al. found no post-surgery infections at discharge in either pre- and post-implementation groups [35].

Few studies included outcomes related to the family members. None reported on incentives for the family to participate in care. Facilitators described to perform the intervention were the use of manuals, instructional videos, posters, handouts and checklists. Both the studies on stroke described no difference in caregiver burden between family-delivered and staff-delivered rehabilitation. Barriers for the family to participate were briefly mentioned in the study of Zhou et al. [31], family work commitments, and Wong et al. [35], language barriers between staff and family.

Very little was reported regarding staff and health service-related outcomes. The reported staff outcomes described the nursing staff’s belief in the benefits of the intervention and initial enthusiasm to engage with the intervention but also described conflicting responsibilities due to a high workload in the long run. Regarding health service outcomes, one study reported the intervention cost as a modest salary for two staff members, but no exact costs were described. Two studies reported staff training as a needed resource to deliver the intervention.

## DISCUSSION

To our knowledge, this systematic review is the first to assess the effect and safety of family participation interventions in resource-limited adult hospital care. The limited and heterogeneous research suggests that family participation, in certain contexts, can positively affect patient outcomes, and none of the studies showed harm. However, at this point, too little data was reported to suggest that family participation can tackle health worker shortages.

To contextualise these findings, studies from resource-rich countries have shown that family participation in staff-delivered care can enhance patient outcomes and lower caregiver burden [36,37]. In paediatrics, family-centred care has been studied more extensively and proven effective in resource-rich and -limited settings [38,39]. Many successful initiatives have been in resource-limited community-based care, often delivered by family or hired lay health care workers with qualified health workers involved remotely [40-44]. Family participation for adults admitted in resource-limited hospitals is inherently different from the settings mentioned above. The family participation described in this review is necessity-driven, shifts tasks from paid health workers to unpaid family members, does not rely on the exclusive parent-child dynamic, encompasses more critical illnesses with complex hospital treatments, poses additional hazards for patients and family, and involves overburdened, understaffed and underfunded health care services.

An interpretation of the scarcity of research, only six studies were included, might be the underdeveloped nature of family participation interventions in resource-limited hospitals. Although, it is probable that more family participation initiatives exist, formal and informal, that have not been described in research. In addition, the high heterogeneity in the study design, participants, intervention, and outcomes highlight the absence of common practice for family participation research and hinder the generalisation of results. For instance, little data were collected on family, staff, and health service-related outcomes to contextualise the findings. On the other hand, the found heterogeneity emphasises the wide range of family participation applications.

Although generalisation from these studies is not possible, they have shown, in specific contexts, that the participation of family members in hospital care can benefit patient outcomes. The difference in success might be attributed to the complexity of the intervention, available support, needed commitment, adherence and maximal effect size if the care task was executed perfectly. For example, the delirium prevention study by Wang et al. involved a simple and proven effective intervention that enabled personalised care by the family for which staff did not have the time. However, this does not explain the absence of effect in the stroke rehabilitation studies of Lindley et al. [30] and Zhou et al. [31]. In this case, the interventions might be too complex, require too much commitment from family and staff, and when executed perfectly, not reach sizable outcomes.

Beyond focusing on positive patient outcomes, finding no harm or difference from involving the family in care can be equally relevant. No harm from family participation was found, suggesting that family participation might be safe in a high-risk environment like a hospital. Additionally, finding no difference in outcomes between care delivered by staff and family might suggest no quality is lost when shifting care from staff to family. Although, in these cases, one might hope to find a positive effect in other domains, such as family, staff, and health service-related outcomes, to justify its implementation. For instance, the lowered burden on staff.

There are several limitations to this review. First, the field of family participation and the associated syntax is largely undefined. Therefore, even with a broad search and reference checks, it is not certain that all eligible studies were found. Second, the geographical spreading of the included studies was poor. It is improbable that these findings represent the spread of family participation needs and are probably biased due to language barriers, underreporting, and lack of local resources for research and implementation. Notably, no studies from South-America were included. Third, the articles were too heterogeneous to pool their data and generalise the findings. Finally, all three non-randomised studies were scored at critical risk of bias, leaving uncertainty on the part family participation played in their findings.

This review’s strengths are its novelty, methods and contextualisation of a new research topic. It is the first to scope the limited literature on family participation interventions and their outcomes in resource-limited adult hospital care. Thus, the PRISMA guidelines and the help of an experienced clinical librarian were used to ensure the correct design and execution in a new field of research. Lastly, the review provides context and recommendations supported by findings, observational studies, and research in related fields.

To serve universal health coverage, future family participation research, preferably clinical trials, should focus on “low cost, but high impact” interventions applicable to a wide range of patients and hospital departments; for instance, patient safety, patient monitoring, medication administration, wound care, nutrition, hygiene or infection prevention. Additionally, the research should consider all involved parties and their outcomes by working in close collaboration and equal partnership with patients, family, staff, and health services. Contextualising the research is needed to reach sizeable outcomes, identify facilitators and barriers for sustainable implementation and generalise the findings to enhance care and tackle health workers’ shortage. Family participation research in resource-limited hospitals should start with the low-hanging fruits.

## CONCLUSIONS

Novel solutions are urgently needed to avert the predicted 18 million health worker deficit in 2030. This systematic review shows that appropriate family participation interventions can be designed to affect patient outcomes positively, and no harm was found from participation. However, little data was found on its effects on the family, staff and health service. More data are needed to determine the effect of family participation in resource-limited hospitals and justify further implementation. Family participation research for enhancing care while tackling health worker shortages should be a collaborative priority of researchers, health care professionals, funding agencies and policymakers.

## Additional material


Online Supplementary Document
