# Supplementary material for: Family participation to enhance care and tackle health worker shortages in resource-limited hospitals: A systematic review
Source: J Glob Health. 2023 Jan 20;13:04005. doi: 10.7189/13.04005 (PMC9853090; doi:10.7189/13.04005)

**ONLINE SUPPLEMENTARY DOCUMENT**

**Title:** Family participation to enhance care and tackle health worker shortages in resource-limited hospitals: A systematic review

**Authors:** Jim J Determeijer, Stije J Leopold, René Spijker, Charles Agyemang & Michèle van Vugt

## Supplement 1

Full search queries performed in Medline, Embase, CINAHL and the Global Health Library on 30-07-2020 and updated on 07-04-2022.

**Medline**

| <u>Keyword</u> | <u>Sub-components</u> | <u>Syntax</u> |
|---|---|---|
| **Resource-limited setting** | | |
| | Developing Countries.sh,kf. | OR |
| | (Africa or Asia or Caribbean or West Indies or South America or Latin America or Central America).hw,kf,ti,ab,cp. | OR |
| | (Afghanistan or Albania or Algeria or Angola or Antigua or Barbuda or Argentina or Armenia or Armenian or Aruba or Azerbaijan or Bahrain or Bangladesh or Barbados or Benin or Byelarus or Byelorussian or Belarus or Belorussian or Belorussia or Belize or Bhutan or Bolivia or Bosnia or Herzegovina or Hercegovina or Botswana or Brasil or Brazil or Bulgaria or Burkina Faso or Burkina Fasso or Upper Volta or Burundi or Urundi or Cambodia or Khmer Republic or Kampuchea or Cameroon or Cameroons or Cameron or Camerons or Cape Verde or Central African Republic or Chad or Chile or China or Colombia or Comoros or Comoro Islands or Comores or Mayotte or Congo or Zaire or Costa Rica or Cote d'Ivoire or Ivory Coast or Croatia or Cuba or Cyprus or Czechoslovakia or Czech Republic or Slovakia or Slovak Republic or Djibouti or French Somaliland or Dominica or Dominican Republic or East Timor or East Timur or Timor Leste or Ecuador or Egypt or United Arab Republic or El Salvador or Eritrea or Estonia or Ethiopia or Fiji or Gabon or Gabonese Republic or Gambia or Gaza or Georgia Republic or Georgian Republic or Ghana or Gold Coast or Greece or Grenada or Guatemala or Guinea or Guam or Guiana or Guyana or Haiti or Honduras or Hungary or India or Maldives or Indonesia or Iran or Iraq or Isle of Man or Jamaica or Jordan or Kazakhstan or Kazakh or Kenya or Kiribati or Korea or Kosovo or Kyrgyzstan or Kirghizia or Kyrgyz Republic or Kirghiz or Kirgizstan or Lao PDR or Laos or Latvia or Lebanon or Lesotho or Basutoland or Liberia or Libya or Lithuania or Macedonia or Madagascar or Malagasy Republic or Malaysia or Malaya or Malay or Sabah or Sarawak or Malawi or Nyasaland or Mali or Malta or Marshall Islands or Mauritania or Mauritius or Agalega Islands or Mexico or Micronesia or Middle East or Moldova or Moldovia or Moldovian or Mongolia or Montenegro or Morocco or Ifni or Mozambique or Myanmar or Myanma or Burma or Namibia or Nepal or Netherlands Antilles or New Caledonia or Nicaragua or Niger or Nigeria or Northern Mariana Islands or Oman or Muscat or Pakistan or Palau or Palestine or Panama or Paraguay or Peru or Philippines or Philipines or Phillipines or Phillippines or Poland or Portugal or Puerto Rico or Romania or Rumania or Roumania or Russia or Russian or Rwanda or Ruanda or Saint Kitts or St Kitts or Nevis or Saint Lucia or St Lucia or Saint Vincent or St Vincent or Grenadines or Samoa or Samoan Islands or Navigator Island or Navigator Islands or Sao Tome or Saudi Arabia or Senegal or Serbia or Montenegro or Seychelles or Sierra Leone or Slovenia or Sri Lanka or Ceylon or Solomon Islands or Somalia or South Africa or Sudan or Suriname or Surinam or Swaziland or Syria or Tajikistan or Tadzhikistan or Tadjikistan or Tadzhik or Tanzania or Thailand or Togo or Togolese Republic or Tonga or Trinidad or Tobago or Tunisia or Turkey or Turkmenistan or Turkmen or Uganda or Ukraine or Uruguay or USSR or Soviet Union or Union of Soviet Socialist Republics or Uzbekistan or Uzbek or Vanuatu or New Hebrides or Venezuela or Vietnam or Viet Nam or West Bank or Yemen or Yugoslavia or Zambia or Zimbabwe or Rhodesia).hw,kf,ti,ab,cp. | OR |
| | ((developing or (less* adj developed) or under-developed or underdeveloped or middle-income or (low* adj income) or underserved or under-served or deprived or poor*) adj (countr* or nation? or population? or world)).ti,ab. | OR |
| | ((developing or less* developed or under developed or underdeveloped or middle income or low* income) adj (economy or economies)).ti,ab. | OR |
| | (low* adj (gdp or gnp or gross domestic or gross national)).ti,ab. | OR |
| | (low adj3 middle adj3 countr*).ti,ab. | OR |
| | (lmic or lmics or third world or lami countr*).ti,ab. | OR |
| | transitional countr*.ti,ab. | OR |
| **Hospital-like environments** | | **AND** |
| | exp "Hospitals"/ or exp "Home Care Services, Hospital-Based"/ or exp "Tertiary Care Centers"/ or exp "Secondary Care Centers"/ or exp Hospices/ or exp "Hospital Departments"/ | OR |

| | | |
|---|---|---|
| | (hospital or ICU or in-hospital or hospice or inpatient* or emergency-department or in-patient or ward or hospice* or ((tertiary or secondary) adj (health or instit* or hospital or center or centre))).ti,ab,kf. | OR |
| **Family** | | **AND** |
| | (exp Family/ or (family or relative* or parent* or child* or informal or lay).ti,ab. | |
| **Caregiver** | | **AND** |
| | (exp Caregivers/ or (caregiver or caregiving or caretaker or caretaking or care-giver or care-giving or care-taker or care-taking).ti,ab.)) or ((family adj2 (participation or engagement or involvement or integrated-care or Delivered-Care)) or ((patient or family) adj2 (centred-care or centered-care)) or PFCC or FCC).ti,ab. | |
| **Exclusion criteria** | | **NOT** |
| | (review or letter or case reports).pt. | |

## Embase

| | |
|---|---|
| 1 | Developing Country.sh. |
| 2 | (Africa or Asia or Caribbean or West Indies or South America or Latin America or Central America).hw,ti,ab,cp. |
| 3 | (Afghanistan or Albania or Algeria or Angola or Antigua or Barbuda or Argentina or Armenia or Armenian or Aruba or Azerbaijan or Bahrain or Bangladesh or Barbados or Benin or Byelarus or Byelorussian or Belarus or Belorussian or Belorussia or Belize or Bhutan or Bolivia or Bosnia or Herzegovina or Hercegovina or Botswana or Brasil or Brazil or Bulgaria or Burkina Faso or Burkina Fasso or Upper Volta or Burundi or Urundi or Cambodia or Khmer Republic or Kampuchea or Cameroon or Cameroons or Cameron or Camerons or Cape Verde or Central African Republic or Chad or Chile or China or Colombia or Comoros or Comoro Islands or Comores or Mayotte or Congo or Zaire or Costa Rica or Cote d'Ivoire or Ivory Coast or Croatia or Cuba or Cyprus or Czechoslovakia or Czech Republic or Slovakia or Slovak Republic or Djibouti or French Somaliland or Dominica or Dominican Republic or East Timor or East Timur or Timor Leste or Ecuador or Egypt or United Arab Republic or El Salvador or Eritrea or Estonia or Ethiopia or Fiji or Gabon or Gabonese Republic or Gambia or Gaza or Georgia Republic or Georgian Republic or Ghana or Gold Coast or Greece or Grenada or Guatemala or Guinea or Guam or Guiana or Guyana or Haiti or Honduras or Hungary or India or Maldives or Indonesia or Iran or Iraq or Isle of Man or Jamaica or Jordan or Kazakhstan or Kazakh or Kenya or Kiribati or Korea or Kosovo or Kyrgyzstan or Kirghizia or Kyrgyz Republic or Kirghiz or Kirgizstan or Lao PDR or Laos or Latvia or Lebanon or Lesotho or Basutoland or Liberia or Libya or Lithuania or Macedonia or Madagascar or Malagasy Republic or Malaysia or Malaya or Malay or Sabah or Sarawak or Malawi or Nyasaland or Mali or Malta or Marshall Islands or Mauritania or Mauritius or Agalega Islands or Mexico or Micronesia or Middle East or Moldova or Moldovia or Moldovian or Mongolia or Montenegro or Morocco or Ifni or Mozambique or Myanmar or Myanma or Burma or Namibia or Nepal or Netherlands Antilles or New Caledonia or Nicaragua or Niger or Nigeria or Northern Mariana Islands or Oman or Muscat or Pakistan or Palau or Palestine or Panama or Paraguay or Peru or Philippines or Philipines or Phillipines or Phillippines or Poland or Portugal or Puerto Rico or Romania or Rumania or Roumania or Russia or Russian or Rwanda or Ruanda or Saint Kitts or St Kitts or Nevis or Saint Lucia or St Lucia or Saint Vincent or St Vincent or Grenadines or Samoa or Samoan Islands or Navigator Island or Navigator Islands or Sao Tome or Saudi Arabia or Senegal or Serbia or Montenegro or Seychelles or Sierra Leone or Slovenia or Sri Lanka or Ceylon or Solomon Islands or Somalia or South Africa or Sudan or Suriname or Surinam or Swaziland or Syria or Tajikistan or Tadzhikistan or Tadjikistan or Tadzhik or Tanzania or Thailand or Togo or Togolese Republic or Tonga or Trinidad or Tobago or Tunisia or Turkey or Turkmenistan or Turkmen or Uganda or Ukraine or Uruguay or USSR or Soviet Union or Union of Soviet Socialist Republics or Uzbekistan or Uzbek or Vanuatu or New Hebrides or Venezuela or Vietnam or Viet Nam or West Bank or Yemen or Yugoslavia or Zambia or Zimbabwe or Rhodesia).hw,ti,ab,cp. |
| 4 | ((developing or less* developed or under developed or underdeveloped or middle income or low* income or underserved or under served or deprived or poor*) adj (countr* or nation? or population? or world)).ti,ab. |
| 5 | ((developing or less* developed or under developed or underdeveloped or middle income or low* income) adj (economy or economies)).ti,ab. |
| 6 | (low* adj (gdp or gnp or gross domestic or gross national)).ti,ab. |
| 7 | (low adj3 middle adj3 countr*).ti,ab. |
| 8 | (lmic or lmics or third world or lami countr*).ti,ab. |
| 9 | transitional countr*.ti,ab. |
| 10 | 1 or 2 or 3 or 4 or 5 or 6 or 7 or 8 or 9 |
| 11 | cancer center/ or hospice/ or exp hospital/ or mental health center/ or rehabilitation center/ or secondary care center/ or tertiary care center/ |
| 12 | (hospital or ICU or in-hospital or hospice or inpatient* or emergency-department or in-patient or ward or hospice* or ((tertiary or secondary) adj (health or instit* or hospital or center or centre))).ti,ab,kw. |
| 13 | 11 or 12 |
| 14 | ((family/ or exp aunt/ or exp cousin/ or extended family/ or exp family relation/ or exp grandchild/ or exp grandparent/ or nephew/ or niece/ or exp stepfamily/ or exp uncle/ or (family or relative* or parent* or child* or informal or lay).ti,ab.) and (Caregiver/ or (caregiver or caregiving or caretaker or caretaking or care-giver or care-giving or care-taker or care-taking).ti,ab.)) or ((family adj2 (participation or engagement or involvement or integrated-care or Delivered-Care)) or ((patient or family) adj2 (centred-care or centered-care)) or PFCC or FCC).ti,ab. |
| 15 | 10 and 13 and 14 |
| 16 | exp "review"/ |
| 17 | exp case report/ |
| 18 | exp letter/ |
| 19 | 16 or 17 or 18 |
| 20 | 15 not 19 |
| 21 | limit 20 to embase |

**CINAHL**

| | |
|---|---|
| 1 | TI(transitional countr*) OR AB(transitional countr*) OR TI(lmic or lmics or third world or lami countr*) OR AB(lmic or lmics or third world or lami countr*) OR TI(low N3 middle N3 countr*) OR AB(low N3 middle N3 countr*) OR TI(low* N1 (gdp or gnp or gross domestic or gross national)) OR AB(low* N1 (gdp or gnp or gross domestic or gross national)) OR TI((developing or less* developed or under developed or underdeveloped or middle income or low* income) N1 (economy or economies)) OR AB((developing or less* developed or under developed or underdeveloped or middle income or low* income) N1 (economy or economies)) OR TI((developing or (less* N1 developed) or under-developed or underdeveloped or middle-income or (low* N1 income) or underserved or under-served or deprived or poor*) N1 (countr* or nation? or population? or world)) OR AB((developing or (less* N1 developed) or under-developed or underdeveloped or middle-income or (low* N1 income) or underserved or under-served or deprived or poor*) N1 (countr* or nation? or population? or world)) OR TI(Afghanistan or Albania or Algeria or Angola or Antigua or Barbuda or Argentina or Armenia or Armenian or Aruba or Azerbaijan or Bahrain or Bangladesh or Barbados or Benin or Byelarus or Byelorussian or Belarus or Belorussian or Belorussia or Belize or Bhutan or Bolivia or Bosnia or Herzegovina or Hercegovina or Botswana or Brasil or Brazil or Bulgaria or Burkina Faso or Burkina Fasso or Upper Volta or Burundi or Urundi or Cambodia or Khmer Republic or Kampuchea or Cameroon or Cameroons or Cameron or Camerons or Cape Verde or Central African Republic or Chad or Chile or China or Colombia or Comoros or Comoro Islands or Comores or Mayotte or Congo or Zaire or Costa Rica or Cote d'Ivoire or Ivory Coast or Croatia or Cuba or Cyprus or Czechoslovakia or Czech Republic or Slovakia or Slovak Republic or Djibouti or French Somaliland or Dominica or Dominican Republic or East Timor or East Timur or Timor Leste or Ecuador or Egypt or United Arab Republic or El Salvador or Eritrea or Estonia or Ethiopia or Fiji or Gabon or Gabonese Republic or Gambia or Gaza or Georgia Republic or Georgian Republic or Ghana or Gold Coast or Greece or Grenada or Guatemala or Guinea or Guam or Guiana or Guyana or Haiti or Honduras or Hungary or India or Maldives or Indonesia or Iran or Iraq or Isle of Man or Jamaica or Jordan or Kazakhstan or Kazakh or Kenya or Kiribati or Korea or Kosovo or Kyrgyzstan or Kirghizia or Kyrgyz Republic or Kirghiz or Kirgizstan or Lao PDR or Laos or Latvia or Lebanon or Lesotho or Basutoland or Liberia or Libya or Lithuania or Macedonia or Madagascar or Malagasy Republic or Malaysia or Malaya or Malay or Sabah or Sarawak or Malawi or Nyasaland or Mali or Malta or Marshall Islands or Mauritania or Mauritius or Agalega Islands or Mexico or Micronesia or Middle East or Moldova or Moldovia or Moldovian or Mongolia or Montenegro or Morocco or Ifni or Mozambique or Myanmar or Myanma or Burma or Namibia or Nepal or Netherlands Antilles or New Caledonia or Nicaragua or Niger or Nigeria or Northern Mariana Islands or Oman or Muscat or Pakistan or Palau or Palestine or Panama or Paraguay or Peru or Philippines or Philipines or Phillipines or Phillippines or Poland or Portugal or Puerto Rico or Romania or Rumania or Roumania or Russia or Russian or Rwanda or Ruanda or Saint Kitts or St Kitts or Nevis or Saint Lucia or St Lucia or Saint Vincent or St Vincent or Grenadines or Samoa or Samoan Islands or Navigator Island or Navigator Islands or Sao Tome or Saudi Arabia or Senegal or Serbia or Montenegro or Seychelles or Sierra Leone or Slovenia or Sri Lanka or Ceylon or Solomon Islands or Somalia or South Africa or Sudan or Suriname or Surinam or Swaziland or Syria or Tajikistan or Tadzhikistan or Tadjikistan or Tadzhik or Tanzania or Thailand or Togo or Togolese Republic or Tonga or Trinidad or Tobago or Tunisia or Turkey or Turkmenistan or Turkmen or Uganda or Ukraine or Uruguay or USSR or Soviet Union or Union of Soviet Socialist Republics or Uzbekistan or Uzbek or Vanuatu or New Hebrides or Venezuela or Vietnam or Viet Nam or West Bank or Yemen or Yugoslavia or Zambia or Zimbabwe or Rhodesia) OR AB(Afghanistan or Albania or Algeria or Angola or Antigua or Barbuda or Argentina or Armenia or Armenian or Aruba or Azerbaijan or Bahrain or Bangladesh or Barbados or Benin or Byelarus or Byelorussian or Belarus or Belorussian or Belorussia or Belize or Bhutan or Bolivia or Bosnia or Herzegovina or Hercegovina or Botswana or Brasil or Brazil or Bulgaria or Burkina Faso or Burkina Fasso or Upper Volta or Burundi or Urundi or Cambodia or Khmer Republic or Kampuchea or Cameroon or Cameroons or Cameron or Camerons or Cape Verde or Central African Republic or Chad or Chile or China or Colombia or Comoros or Comoro Islands or Comores or Mayotte or Congo or Zaire or Costa Rica or Cote d'Ivoire or Ivory Coast or Croatia or Cuba or Cyprus or Czechoslovakia or Czech Republic or Slovakia or Slovak Republic or Djibouti or French Somaliland or Dominica or Dominican Republic or East Timor or East Timur or Timor Leste or Ecuador or Egypt or United Arab Republic or El Salvador or Eritrea or Estonia or Ethiopia or Fiji or Gabon or Gabonese Republic or Gambia or Gaza or Georgia Republic or Georgian Republic or Ghana or Gold Coast or Greece or Grenada or Guatemala or Guinea or Guam or Guiana or Guyana or Haiti or Honduras or Hungary or India or Maldives or Indonesia or Iran or Iraq or Isle of Man or Jamaica or Jordan or Kazakhstan or Kazakh or Kenya or Kiribati or Korea or Kosovo or Kyrgyzstan or Kirghizia or Kyrgyz Republic or Kirghiz or Kirgizstan or Lao PDR or Laos or Latvia or Lebanon or Lesotho or Basutoland or Liberia or Libya or Lithuania or Macedonia or Madagascar or Malagasy Republic or Malaysia or Malaya or Malay or Sabah or Sarawak or Malawi or Nyasaland or Mali or Malta or Marshall Islands or Mauritania or Mauritius or Agalega Islands or Mexico or Micronesia or Middle East or Moldova or Moldovia or Moldovian or Mongolia or Montenegro or Morocco or Ifni or Mozambique or Myanmar or Myanma or Burma or Namibia or Nepal or Netherlands Antilles or New Caledonia or Nicaragua or Niger or Nigeria or Northern Mariana Islands or Oman or Muscat or Pakistan or Palau or Palestine or Panama or Paraguay or Peru or Philippines or Philipines or Phillipines or Phillippines or Poland or Portugal or Puerto Rico or Romania or Rumania or Roumania or Russia or Russian or Rwanda or Ruanda or Saint Kitts or St Kitts or Nevis or Saint Lucia or St Lucia or Saint Vincent or St Vincent or Grenadines or Samoa or Samoan Islands or Navigator Island or Navigator Islands or Sao Tome or Saudi Arabia or Senegal or Serbia or Montenegro or Seychelles or Sierra Leone or Slovenia or Sri Lanka or Ceylon or Solomon Islands or Somalia or South Africa or Sudan or Suriname or Surinam or Swaziland or Syria or Tajikistan or Tadzhikistan or Tadjikistan or Tadzhik or Tanzania or Thailand or Togo or Togolese Republic or Tonga or Trinidad or Tobago or Tunisia or Turkey or Turkmenistan or Turkmen or Uganda or Ukraine or Uruguay or USSR or Soviet Union or Union of Soviet Socialist Republics or Uzbekistan or Uzbek or Vanuatu or New Hebrides or Venezuela or Vietnam or Viet Nam or West Bank or Yemen or Yugoslavia or Zambia or Zimbabwe or Rhodesia) OR AF(Afghanistan or Albania or Algeria or Angola or Antigua or Barbuda or Argentina or Armenia or Armenian or Aruba or Azerbaijan or Bahrain or Bangladesh or Barbados or Benin or Byelarus or Byelorussian or Belarus or Belorussian or Belorussia or Belize or Bhutan or Bolivia or Bosnia or Herzegovina or Hercegovina or Botswana or Brasil or Brazil or Bulgaria or Burkina Faso or Burkina Fasso or Upper Volta or Burundi or Urundi or Cambodia or Khmer Republic or Kampuchea or Cameroon or Cameroons or Cameron or Camerons or Cape Verde or Central African Republic or Chad or Chile or China or Colombia or Comoros or Comoro Islands or Comores or Mayotte or Congo or Zaire or Costa Rica or Cote d'Ivoire or Ivory Coast or Croatia or Cuba or Cyprus or Czechoslovakia or Czech Republic or Slovakia or Slovak Republic or Djibouti or French Somaliland or Dominica or Dominican Republic or East Timor or East Timur or Timor Leste or Ecuador or Egypt or United Arab Republic or El Salvador or Eritrea or Estonia or Ethiopia or Fiji or Gabon or Gabonese Republic or Gambia or Gaza or Georgia Republic or Georgian Republic or Ghana or Gold Coast or Greece or Grenada or Guatemala or Guinea or Guam or Guiana or Guyana or Haiti or Honduras or Hungary or India or Maldives or Indonesia or Iran or Iraq or Isle of Man or Jamaica or Jordan or Kazakhstan or Kazakh or Kenya or Kiribati or Korea or Kosovo or Kyrgyzstan or Kirghizia or Kyrgyz Republic or Kirghiz or Kirgizstan or Lao PDR or Laos or |

| | |
|---|---|
| | Latvia or Lebanon or Lesotho or Basutoland or Liberia or Libya or Lithuania or Macedonia or Madagascar or Malagasy Republic or Malaysia or Malaya or Malay or Sabah or Sarawak or Malawi or Nyasaland or Mali or Malta or Marshall Islands or Mauritania or Mauritius or Agalega Islands or Mexico or Micronesia or Middle East or Moldova or Moldovia or Moldovian or Mongolia or Montenegro or Morocco or Ifni or Mozambique or Myanmar or Myanma or Burma or Namibia or Nepal or Netherlands Antilles or New Caledonia or Nicaragua or Niger or Nigeria or Northern Mariana Islands or Oman or Muscat or Pakistan or Palau or Palestine or Panama or Paraguay or Peru or Philippines or Philipines or Phillipines or Phillippines or Poland or Portugal or Puerto Rico or Romania or Rumania or Roumania or Russia or Russian or Rwanda or Ruanda or Saint Kitts or St Kitts or Nevis or Saint Lucia or St Lucia or Saint Vincent or St Vincent or Grenadines or Samoa or Samoan Islands or Navigator Island or Navigator Islands or Sao Tome or Saudi Arabia or Senegal or Serbia or Montenegro or Seychelles or Sierra Leone or Slovenia or Sri Lanka or Ceylon or Solomon Islands or Somalia or South Africa or Sudan or Suriname or Surinam or Swaziland or Syria or Tajikistan or Tadzhikistan or Tadjikistan or Tadzhik or Tanzania or Thailand or Togo or Togolese Republic or Tonga or Trinidad or Tobago or Tunisia or Turkey or Turkmenistan or Turkmen or Uganda or Ukraine or Uruguay or USSR or Soviet Union or Union of Soviet Socialist Republics or Uzbekistan or Uzbek or Vanuatu or New Hebrides or Venezuela or Vietnam or Viet Nam or West Bank or Yemen or Yugoslavia or Zambia or Zimbabwe or Rhodesia) OR KW(Afghanistan or Albania or Algeria or Angola or Antigua or Barbuda or Argentina or Armenia or Armenian or Aruba or Azerbaijan or Bahrain or Bangladesh or Barbados or Benin or Byelarus or Byelorussian or Belarus or Belorussian or Belorussia or Belize or Bhutan or Bolivia or Bosnia or Herzegovina or Hercegovina or Botswana or Brasil or Brazil or Bulgaria or Burkina Faso or Burkina Fasso or Upper Volta or Burundi or Urundi or Cambodia or Khmer Republic or Kampuchea or Cameroon or Cameroons or Cameron or Camerons or Cape Verde or Central African Republic or Chad or Chile or China or Colombia or Comoros or Comoro Islands or Comores or Mayotte or Congo or Zaire or Costa Rica or Cote d'Ivoire or Ivory Coast or Croatia or Cuba or Cyprus or Czechoslovakia or Czech Republic or Slovakia or Slovak Republic or Djibouti or French Somaliland or Dominica or Dominican Republic or East Timor or East Timur or Timor Leste or Ecuador or Egypt or United Arab Republic or El Salvador or Eritrea or Estonia or Ethiopia or Fiji or Gabon or Gabonese Republic or Gambia or Gaza or Georgia Republic or Georgian Republic or Ghana or Gold Coast or Greece or Grenada or Guatemala or Guinea or Guam or Guiana or Guyana or Haiti or Honduras or Hungary or India or Maldives or Indonesia or Iran or Iraq or Isle of Man or Jamaica or Jordan or Kazakhstan or Kazakh or Kenya or Kiribati or Korea or Kosovo or Kyrgyzstan or Kirghizia or Kyrgyz Republic or Kirghiz or Kirgizstan or Lao PDR or Laos or Latvia or Lebanon or Lesotho or Basutoland or Liberia or Libya or Lithuania or Macedonia or Madagascar or Malagasy Republic or Malaysia or Malaya or Malay or Sabah or Sarawak or Malawi or Nyasaland or Mali or Malta or Marshall Islands or Mauritania or Mauritius or Agalega Islands or Mexico or Micronesia or Middle East or Moldova or Moldovia or Moldovian or Mongolia or Montenegro or Morocco or Ifni or Mozambique or Myanmar or Myanma or Burma or Namibia or Nepal or Netherlands Antilles or New Caledonia or Nicaragua or Niger or Nigeria or Northern Mariana Islands or Oman or Muscat or Pakistan or Palau or Palestine or Panama or Paraguay or Peru or Philippines or Philipines or Phillipines or Phillippines or Poland or Portugal or Puerto Rico or Romania or Rumania or Roumania or Russia or Russian or Rwanda or Ruanda or Saint Kitts or St Kitts or Nevis or Saint Lucia or St Lucia or Saint Vincent or St Vincent or Grenadines or Samoa or Samoan Islands or Navigator Island or Navigator Islands or Sao Tome or Saudi Arabia or Senegal or Serbia or Montenegro or Seychelles or Sierra Leone or Slovenia or Sri Lanka or Ceylon or Solomon Islands or Somalia or South Africa or Sudan or Suriname or Surinam or Swaziland or Syria or Tajikistan or Tadzhikistan or Tadjikistan or Tadzhik or Tanzania or Thailand or Togo or Togolese Republic or Tonga or Trinidad or Tobago or Tunisia or Turkey or Turkmenistan or Turkmen or Uganda or Ukraine or Uruguay or USSR or Soviet Union or Union of Soviet Socialist Republics or Uzbekistan or Uzbek or Vanuatu or New Hebrides or Venezuela or Vietnam or Viet Nam or West Bank or Yemen or Yugoslavia or Zambia or Zimbabwe or Rhodesia) OR TI(Africa or Asia or Caribbean or West Indies or South America or Latin America or Central America) Or (Africa or Asia or Caribbean or West Indies or South America or Latin America or Central America) OR KW(Africa or Asia or Caribbean or West Indies or South America or Latin America or Central America) |
| 2 | KW(hospital or ICU or in-hospital or hospice or inpatient* or emergency-department or in-patient or ward or hospice* or ((tertiary or secondary) adj (health or instit* or hospital or center or centre))) OR AB(hospital or ICU or in-hospital or hospice or inpatient* or emergency-department or in-patient or ward or hospice* or ((tertiary or secondary) N1 (health or instit* or hospital or center or centre))) OR TI(hospital or ICU or in-hospital or hospice or inpatient* or emergency-department or in-patient or ward or hospice* or ((tertiary or secondary) N1 (health or instit* or hospital or center or centre))) OR (MH "Hospitals+") OR (MH "Academic Medical Centers") OR (MH "Alternative Health Facilities") OR (MH "Health Facility Departments+") OR (MH "Rehabilitation Centers+") OR (MH "Rural Health Centers") |
| 3 | (AB(family or relative* or parent* or child* or informal or lay) OR TI(family or relative* or parent* or child* or informal or lay) OR (MH "Family+"))AND (AB(caregiver* or caregiving or caretaker* or caretaking or care-giver* or care-giving or care-taker* or care-taking) OR TI(caregiver or caregiving or caretaker or caretaking or care-giver or care-giving or care-taker or care-taking) OR (MH "Caregivers")) |
| 4 | AB((family N2 (participation or engagement or involvement or integrated-care or Delivered-Care)) or ((patient or family) N2 (centred-care or centered-care)) or PFCC or FCC) OR TI((family N2 (participation or engagement or involvement or integrated-care or Delivered-Care)) or ((patient or family) N2 (centred-care or centered-care)) or PFCC or FCC) |
| 5 | S3 OR S4 |
| 6 | S1 AND S2 AND S5 |

## The Global Health Library

| |
|---|
| (ab:((family OR relative* OR informal OR lay-people or lay-individuals) AND (caregiver* OR caregiving OR caretaker* OR caretaking OR care-giver* OR care-giving OR care-taker* OR care-taking) AND (hospital OR in-hospital OR hospice OR inpatient* OR emergency-department OR in-patient OR ward OR hospice*))) OR (ti:((family OR relative* OR informal OR lay-people or lay-individuals) AND (caregiver* OR caregiving OR caretaker* OR caretaking OR care-giver* OR care-giving OR care-taker* OR care-taking) AND (hospital OR in-hospital OR hospice OR inpatient* OR emergency-department OR in-patient OR ward OR hospice*))) |

## Supplement 2

List of excluded studies during full-text screening including reasons.

| Author | Publication year | Title | Exclusion reason |
|---|---|---|---|
| Afaf Mahmoud, F. | 2005 | Dementia: nursing intervention for family care givers | No full-text |
| Akkuzu, G. | 2009 | Evaluation by patients and caregivers of the effectiveness of a brochure developed to prevent pressure ulcers | No family participation intervention |
| Alibeigi, N. | 2018 | The effectiveness of family-based intervention on symptom severity, expressed emotion and coping styles of bipolar patients | No family participation intervention |
| Al Mutairi, A. | 2020 | Hospital-acquired pressure ulcer incident rates amonghospitals that implement an education program for staff,patients, and family caregivers inclusive of an afterdischarge follow-up program in Saudi Arabia | Not in a LMIC |
| Anh, N. | 2021 | Feasibility of establishing a rehabilitation programme in a Vietnamese intensive care unit | No patient outcome |
| Bahrami, M. | 2014 | The effect of a supportive educational program based on COPE model on caring burden and quality of life in family caregivers of women with breast cancer | No patient outcome |
| Budiono, W. | 2021 | Psychoeducation Improved Illness Perception and Expressed Emotion of Family Caregivers of Patients with Schizophrenia | No family participation intervention |
| Chen, Y. | 2017 | Family Caregiver Contribution to Self-care of Heart Failure: An Application of the Information-Motivation-Behavioral Skills Model | No family participation intervention |
| Cheng, L. | 2005 | Psychoeducation program for Chinese family carers of members with schizophrenia | Not in a LMIC |
| Cho, B. | 2011 | Effects of Video-guided Education for Primary Family-caregivers of Stroke Patients | No English, French or Dutch |
| Cho, K. | 2007 | Effects of an Information Protocol on Anxiety and Nursing Satisfaction for Family Caregivers of Cerebrovascular Accident Patients in the ICU | No English, French or Dutch |
| Chu. K | 2020 | Feasibility of a Nurse-Trained, Family Member-Delivered Rehabilitation Model for Disabled Stroke Patients in Rural Chongqing, China | No participation in a hospital-like environment |
| Deek, H. | 2017 | An evaluation of involving family caregivers in the self-care of heart failure patients on hospital readmission: Randomised controlled trial (the FAMILY study) | No participation in a hospital-like environment |
| Deek, H. | 2020 | A single educational intervention on heart failure self-care: Extended follow-up from a multisite randomized controlled trial | No participation in a hospital-like environment |
| Deyhoul, N. | 2018 | The Effect of Educational Intervention on Family Caregivers' Perceived Threat of the Risk of Dependence Among Patients with Stroke: A Randomized Clinical Trial | No participation in a hospital-like environment |
| Ebrahimi, H. | 2018 | Effect of Family-Based Education on the Quality of Life of Persons with Type 2 Diabetes: A Randomized Clinical Trial | No participation in a hospital-like environment |
| Eskes, A. | 2019 | Developing an evidence-based and theory informed intervention to involve families in patients care after surgery: A quality improvement project | No family participation intervention |
| Etemadifar, S. | 2014 | The effectiveness of a supportive educative group intervention on family caregiver burden of patients with heart failure | No patient outcome |
| Fathani, M. | 2016 | Evaluation of the effect of designated educational intervention on the improvement of quality of life in caregivers of patients with chronic heart failure | No English, French or Dutch |
| Flores, Y. | 2010 | Teaching the caregiver the art of caring: an educational care program with multiple trauma patient caregivers | No English, French or Dutch |
| García-Cardoza, I. | 2018 | Efectos de la terapia cognitivo-conductual en la sobrecarga del cuidador primario de adultos mayores | No English, French or Dutch |
| Gonçalves, J. | 2010 | Assistence to family caregivers of patients suffering from mental disorder | No English, French or Dutch |
| Hekmatpou, D. | 2019 | The effect of patient care education on burden of care and the quality of life of caregivers of stroke patients | No patient outcome |
| Hong, S. | 2017 | Effect of a caregiver's education program on stroke rehabilitation | Not in a LMIC |
| Ildarabadi, E. | 2019 | Comparison of face-to-face education of caregivers and hospitalized elderlies with copd and its impact on the elderlies' self-care | No participation in a hospital-like environment |
| Ip, S. | 2004 | Short-stay in-patient rehabilitation of elderly patients with chronic obstructive pulmonary disease: Prospective study | Not in a LMIC |
| Jahangiri, M. | 2016 | Effect of family centered care on patient's family satisfaction in intensive care unit | No family participation intervention |

| | | | |
|---|---|---|---|
| Jinglian, C. | 2019 | Effect of transitional care based on IMB model in hematological neoplasms caregivers | No full-text |
| Kang, K. | 2005 | Evaluation of a Self-efficacy-based Basic Life Support Program for High-risk Patients' Family Caregivers | No English, French or Dutch |
| Kang, Y. | 2017 | South Korean family caregiver: Involvement in delirium care a qualitative descriptive study | Not in a LMIC |
| Kang, Y. | 2017 | An educational programme to improve acute care nurses' knowledge, attitudes and family caregiver involvement in care of people with cognitive impairment | Not in a LMIC |
| Kim, B. | 2000 | The Effect of Support Group Intervention on Various Adaptations of Primary Family Caregivers Caring for Cerebro-Vascular Accident Patients | No English, French or Dutch |
| Kim, E. | 2010 | Hands-on Training for Caregivers of Post-Stroke Inpatients | No English, French or Dutch |
| Kim, H. | 2016 | The effect of patient-centered CPR education for family caregivers of patients with cardiovascular diseases | Not in a LMIC |
| Koohi, M. | 2017 | Effect of family participation in primary care provision to reduce pain anxiety among burn ICU patients | No English, French or Dutch |
| Madhanraj, K. | 2019 | Impact of self-instruction manual-based training of family caregivers of neurosurgery patients on their knowledge and care practices - A randomized controlled trial | No patient outcome |
| Michael, N. | 2015 | A mixed method feasibility study of a patient- and family-centred advance care planning intervention for cancer patients | No family participation intervention |
| Moieni, M. | 2014 | Investigating the effect of family-focused nursing intervention on caregiver burden of the family members of the patients undergoing coronary bypass surgery in Isfahan Shahid Chamran Hospital during 2012 | No patient outcome |
| Mudzi, W. | 2012 | Effect of carer education on functional abilities of patients with stroke | No participation in a hospital-like environment |
| Nattala, P. | 2010 | Family member involvement in relapse prevention improves alcohol dependence outcomes: A prospective study at an addiction treatment facility in India | No family participation intervention |
| Nkhoma, K. | 2013 | An educational intervention to reduce pain and improve pain management for Malawian people living with HIV/AIDS and their family carers: Study protocol for a randomised controlled trial | Wrong study type |
| Oupra, R. | 2010 | Effectiveness of Supportive Educative Learning programme on the level of strain experienced by caregivers of stroke patients in Thailand | No patient outcome |
| Ozkan, B. | 2013 | Effect of psychoeducation and telepsychiatric follow up given to the caregiver of the schizophrenic patient on family burden, depression and expression of emotion | No patient outcome |
| Pandian, J. | 2015 | FAmily-Led RehabiliTaTion aftEr Stroke in INDia: The ATTEND pilot study | No patient outcome |
| Qingrong, Q. | 2019 | Effects of family caregivers'cognitive behavioral intervention on nutritional status of postoperative gastric cancer patients during chemotherapy | No full-text |
| Roy, M. | 2019 | Effectiveness of Structured Teaching Programme on the Knowledge about Rehabilitation of Stroke Patients among Caregivers in a Selected Hospital, Kolkata, India | No patient outcome |
| Rwashana, S. | 1994 | Toward a more friendly social environment for the patient: the role of the resident hospital relative | No full-text |
| Santos, N. | 2020 | Development and validation a nursing care protocol with educational interventions for family caregivers of elderly people after stroke | No family participation intervention |
| Senanarong, V. | 2004 | A counseling intervention for caregivers: Effect on neuropsychiatric symptoms | No participation in a hospital-like environment |
| Shamsaei, F. | 2018 | The effect of training interventions of stigma associated with mental illness on family caregivers: A quasi-experimental study 11 Medical and Health Sciences 1117 Public Health and Health Services | No family participation intervention |
| Souaibi, L. | 2016 | Therapeutic education of families of patients with schizophrenia: The Lebanese Hôtel-Dieu de France model | No participation in a hospital-like environment |
| Thimmajja, S. | 2019 | Effectiveness of psycho-education on knowledge regarding schizophrenia and caregivers' burden among caregivers of patients with schizophrenia – A randomized controlled trial | No patient outcome |
| Tzeng, H. | 2007 | Using Family Visitors, Sitters, or Volunteers to Prevent Inpatient Falls | No family participation intervention |
| Worakul, P. | 2007 | Effects of psycho-educational program on knowledge and attitude upon schizophrenia of schizophrenic patients' caregivers | No patient outcome |
| Xiaoli, W. | 2017 | Application of WeChat public platform on continuous nursing for patients with high risk of pressure ulcers | No full-text |

| | | | |
|---|---|---|---|
| Xin, L. | 2016 | Evaluation of the intervention from wound expert on the primary caregivers' behavior of caring ;patients with pressure ulcers | No full-text |
| Xiong, W. | 1994 | Family-based intervention for schizophrenic patients in China. A randomised controlled trial | No participation in a hospital-like environment |
| Xu, Z. | 2018 | The effects of family empowerment program on comprehensive care ability and care preparedness for main caregivers of patients with initial stroke | No full-text |
| Yan, L. | 2016 | A randomized controlled trial on rehabilitation through caregiver-delivered nurse-organized service programs for disabled stroke patients in rural china (the RECOVER trial): design and rationale | Wrong study type |
| Yanqing, T. | 2017 | Observation on the application of family nursing model based on the timing theory in acute myocardial infarction | No full-text |
| Yoo, E. | 2007 | The effects of a support group intervention on the burden of primary family caregivers of stroke patients | No English, French or Dutch |
| Yu, Y. | 2020 | Assessing a WeChat-based integrative family intervention (WIFI) for schizophrenia: protocol for a stepped-wedge cluster randomized trial (Preprint) | Wrong study type |
| Yulin, X. | 2021 | Influence of empowerment intervention mode of family caregivers on discharge preparation and quality of life of lung cancer patients undergoing chemotherapy | No full-text |

## Supplement 3

| Overview of the reported predefined data items within the included studies | | | | | | | |
|---|---|---|---|---|---|---|---|
| | **Lindley et al.** | **Zhou et al.** | **Wang et al.** | **Kristanti et al.** | **Alupo et al.** | **Wong et al.** | **Total** |
| Patient outcomes | | | | | | | |
| Mortality | X | | X | | | | **2** |
| Adverse events | X | | | | | X | **2** |
| Length of stay | X | X | X | | | | **3** |
| Readmission | X | | | | | | **1** |
| Disability and dependence | X | X | | | | | **2** |
| Effect on symptoms | X | X | X | X | X | | **5** |
| Quality of life | X | X | | X | | | **3** |
| Family outcomes | | | | | | | |
| Incentives | | | | | | | **0** |
| Facilitators | X | X | | | X | X | **4** |
| Burden | X | X | | | | | **2** |
| Barriers | | X | | | | X | **2** |
| Staff outcomes | | | | | | | |
| Staff perception | | X | | | | X | **2** |
| Staff burden | | X | | | X | X | **3** |
| Health service outcomes | | | | | | | |
| Cost of intervention | | | | | X | | **1** |
| Hospital resources | | X | X | | | | **2** |

## Supplement 4

Risk of Bias: ROB2 results visualised with the ROBVIS tool

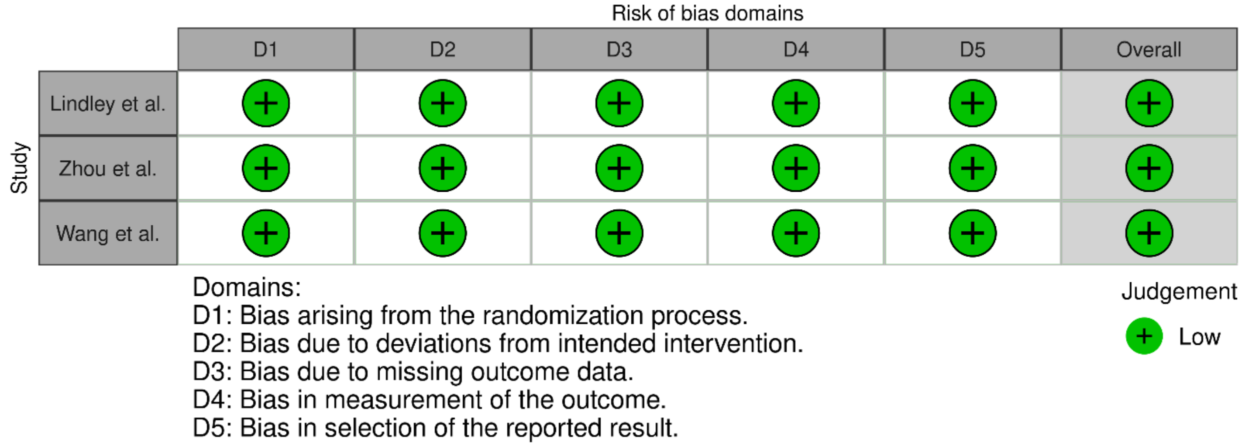


Risk of Bias: ROBINS-I results visualised with the ROBVIS tool

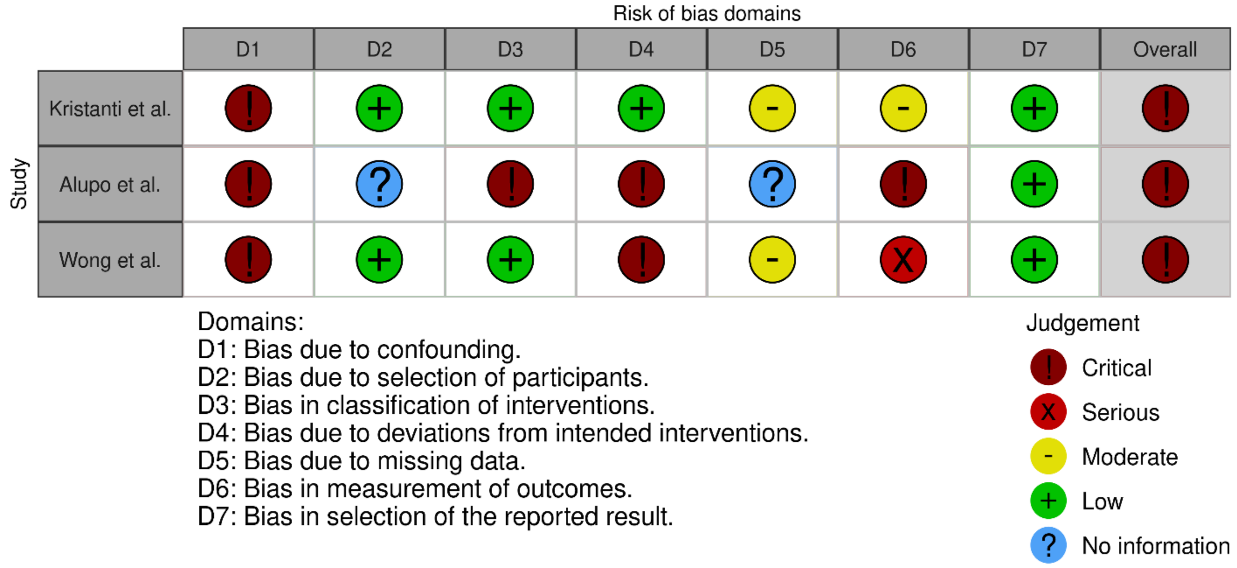

Supplement: Online Supplementary Document [file jogh-13-04005-s001.pdf]
